# Tracing the Origins of IgE, Mast Cells, and Allergies by Studies of Wild Animals

**DOI:** 10.3389/fimmu.2017.01749

**Published:** 2017-12-19

**Authors:** Lars Torkel Hellman, Srinivas Akula, Michael Thorpe, Zhirong Fu

**Affiliations:** ^1^Department of Cell and Molecular Biology, Uppsala University, Uppsala, Sweden

**Keywords:** IgE, Fc receptor, mast cell, IgE homeostasis, allergy, dermatitis, asthma

## Abstract

In most industrialized countries, allergies have increased in frequency quite dramatically during the past 50 years. Estimates show that 20–30% of the populations are affected. Allergies have thereby become one of the major medical challenges of the twenty-first century. Despite several theories including the hygiene hypothesis, there are still very few solid clues concerning the causes of this increase. To trace the origins of allergies, we have studied cells and molecules of importance for the development of IgE-mediated allergies, including the repertoire of immunoglobulin genes. These studies have shown that IgE and IgG most likely appeared by a gene duplication of IgY in an early mammal, possibly 220–300 million years ago. Receptors specific for IgE and IgG subsequently appeared in parallel with the increase in Ig isotypes from a subfamily of the recently identified Fc receptor-like molecules. Circulating IgE levels are generally very low in humans and laboratory rodents. However, when dogs and Scandinavian wolfs were analyzed, IgE levels were found to be 100–200 times higher compared to humans, indicating a generally much more active IgE synthesis in free-living animals, most likely connected to intestinal parasite infections. One of the major effector molecules released upon IgE-mediated activation by mast cells are serine proteases. These proteases, which belong to the large family of hematopoietic serine proteases, are extremely abundant and can account for up to 35% of the total cellular protein. Recent studies show that several of these enzymes, including the chymases and tryptases, are old. Ancestors for these enzymes were most likely present in an early mammal more than 200 million years ago before the separation of the three extant mammalian lineages; monotremes, marsupials, and placental mammals. The aim is now to continue these studies of mast cell biology and IgE to obtain additional clues to their evolutionary conserved functions. A focus concerns why the humoral immune response involving IgE and mast cells have become so dysregulated in humans as well as several of our domestic companion animals.

## Introduction

During the past 50 years, allergies has increased in prevalence quite dramatically and in most industrialized countries 20–30% of the population are affected. In some school classes, the percentage of affected children can reach as high as 50. Allergies have thereby become one of the major medical challenges of the twenty-first century. Although relatively few die from an anaphylactic shock, the severest form of allergic reaction, there are extensive burdens for sufferers, which in turn can also cause major economic loss. Asthma in children has in the USA been estimated to cost 56 billion $ per year and asthma in adults to cost 19 billion € per year in the EU so allergies involves large costs for the society (1, 2). Allergies not only affect humans but also our close domestic companions, including dogs, cats, and horses. In dogs, 3–15%, depending on the breed, suffer from atopic dermatitis, a type of allergic skin disease (3–5). By contrast, cats and horses suffer primarily from asthma. This indicates that domestication may be one contributing factor in this process. Rodents are the most frequently used animal models in allergy research and the numerous inbred strains of mice and rats may be seen as a form of domestication. However, neither rats nor mice can be considered naturally allergic. To obtain allergic mice or rats, these animals needs to be triggered by relatively high allergen doses and often complimented with additional immune stimulators to show allergy-like symptoms (6–8). These symptoms also disappear as soon as the sensitization protocol is terminated. Therefore, a life under controlled conditions with low pathogen load does not necessarily result in the induction of hypersensitivity. However, western lifestyle, with high hygiene levels, has been indicated as a potential contributing factor to the increase in allergy prevalence and been termed the “hygiene hypothesis” (9–11). The contact with high levels of bacteria, viruses, and parasites has likely been the normal condition for our immune system during the past millions of years of evolution (12). This has been an important component for the immune system to become educated and if we remove the normal triggers in our daily life, we may be more likely to develop allergies. There is evidence supporting this hypothesis but there is also data that do not fit into this model. One previously mentioned example is rodents living under controlled conditions in animal houses. There are certainly other contributing factors of major importance. Therefore in order to look deeper into these questions, we have turned our interest to the components of our immune system that are involved in an allergic immune response. This approach can be used to see if by studying of an array of different wild, domestic, and non-domestic animals, it is possible to trace the origins of these components and the factors that have resulted in the massive increase in allergies in humans and other domestic animals.

## The Induction of an Allergic Immune Response

The absolute majority of allergies in humans belong to the immunoglobulin (Ig) E-mediated allergies, which also are named atopic allergies. The major focus of this review will be on this type of allergy, where dendritic cells, IgE, and IgE-binding cells, primarily mast cells, and basophils are central players (13).

**Figure 1:**
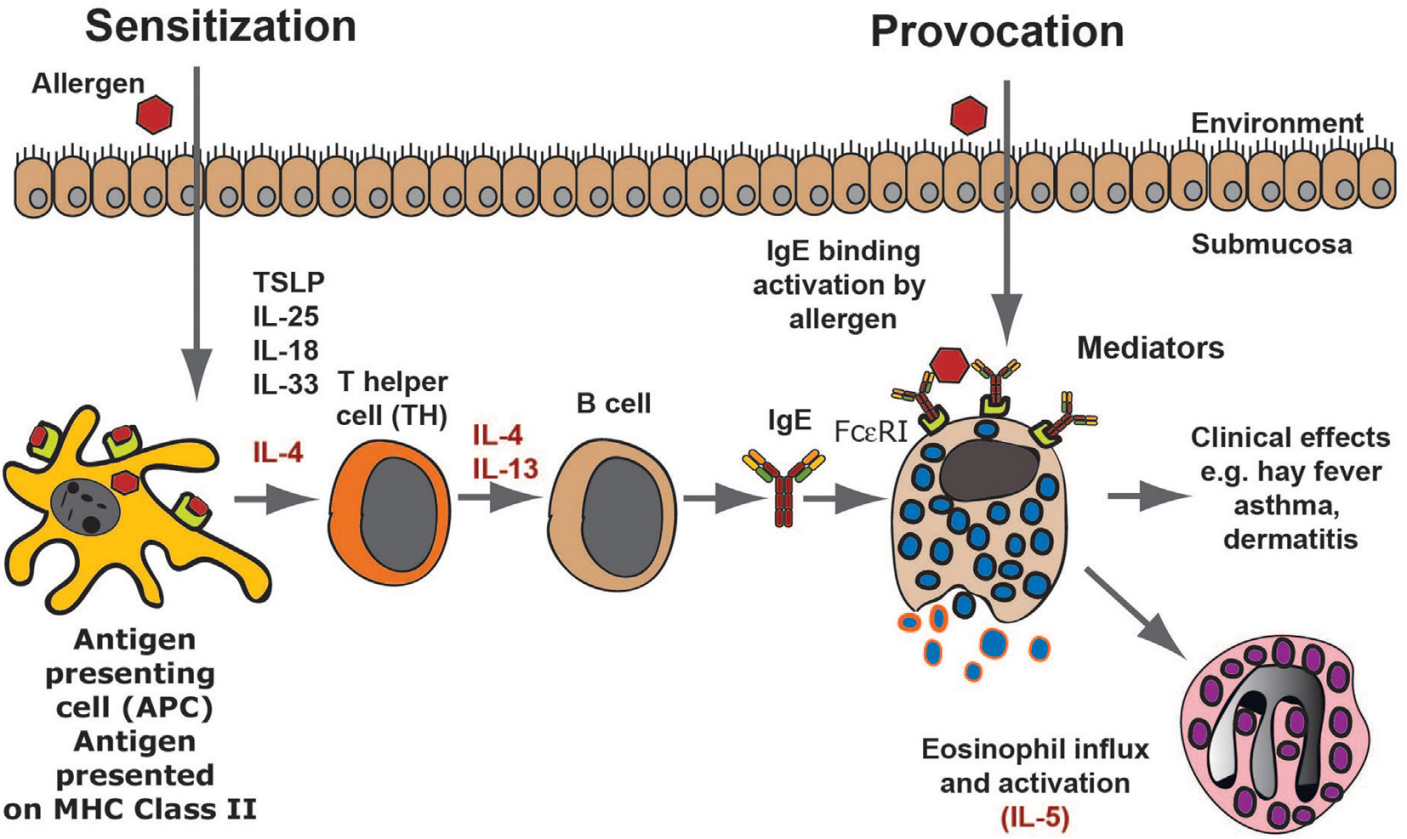
An allergic immune response. The figure presents a schematic overview of an allergic immune response starting with the allergens first contact when it enters through the skin, lungs, or intestinal mucosa. Antigen-presenting cells, primarily dendritic cells, take up the antigen and process it into peptides, which are subsequently presented onto major histocompatibility complex class II molecules to naïve T cells. In an environment lacking major danger signals and in the presence of certain TH2-promoting cytokines, the T cell becomes a TH2 type of T cell. These T cells produce IL-4 and IL-13, which stimulates B cells to switch to IgE production. The IgE produced by these local B cells binds tissue mast cells, which have now become sensitized and can respond by degranulation as well as prostaglandin and leukotriene synthesis, which provides all the symptoms of an allergic reaction. As a next step, locally produced IL-5 results in eosinophil influx and the induction of a late phase response.

The first time we are exposed to an allergen there will be no visible response. However, the immune system may start to recognize the target molecule *via* uptake of the protein antigen, the allergen, by local dendritic cells. Generally, allergens are proteins of a relatively low molecular weight and are often also relatively stable proteins, indicating they can more easily enter the mucosa in a native conformation. Dendritic cells are abundant at sites where allergens generally enter the body, for example, the skin, the lung, or the intestinal mucosa. These cells then process some of the protein into peptides of variable length in endosomal compartments using lysosomal proteases. The peptides, generally between 12 and 18 amino acids, are then presented onto major histocompatibility complex (MHC) class II molecules on the cell surface of the dendritic cell (Figure 1). A T helper cell may then recognize this peptide MHC complex with its specific T cell receptor (TCR) and when supported by several additional receptor ligand interactions, including CD28-B7:1 or B7:2, CD40L–CD40, CD48R–CD22, CD2–LFA3, the T cell becomes activated and starts to proliferate. Furthermore, the activated cell produces cytokines and also to upregulate receptors and receptor ligands that can support and trigger other immune cells (Figure 1). If there is no intracellular parasite or other potent danger signal in the area of allergen entry, the T cells are generally becoming T-helper cells of type 2, so called TH2 cells. TH2 cells promote humoral immunity, consisting primarily of soluble factors, involving B cells and Igs and not in cell-mediated immunity where cytotoxic T cells (CTLs) and NK cells are involved. The TH2 cells produce cytokines, including IL-4, IL-13, IL-5, IL-10, and sometimes also TNF-α (Figure 1) (14, 15). The B cells in the area of allergen contact bind with their surface IgM to the native protein antigen and thereby receive signal 1 to become activated. These B cells are then triggered by local IL-4 and/or IL-13 to switch to IgE and IgG1 producing cells in mice, and IgE and IgG4 in humans (Figure 1) (16–18). The locally produced IgE can then bind to high-affinity receptors on cells in the tissue. The cells that express the high-affinity receptor for IgE are primarily mast cells and basophilic granulocytes (Figure 1). Both of these cells store histamine and can also produce potent lipid mediators including prostaglandins and leukotrienes, which when released from the cells give the characteristic symptoms of allergies including tissue swelling, broncho constriction, and drop in blood pressure. The latter effects occur if mast cell activation involves larger regions of the body. This entire process, from the first antigen contact to the development of IgE and mast cell bound IgE, is termed sensitization and this can take weeks to months or even years to develop (13). In most cases, the immune system regulates itself so that more IgG than IgE is produced meaning no hypersensitivity appears and the individual does not become allergic (Figure 1). However, if this is dysregulated and more IgE is produced, the tissue becomes overly sensitive to contact with the allergen. When a sensitized person comes in contact with the same subsequent allergen, the mast cells release a number of vasoactive substances, which results in tissue swelling due to the influx of liquid into the tissue from the blood. The activation of mast cells also results in the recruitment of eosinophils to the tissue, a process at least partly dependent on locally produced IL-5 (Figure 1).

## Mast Cells and Basophils

**Figure 2:**
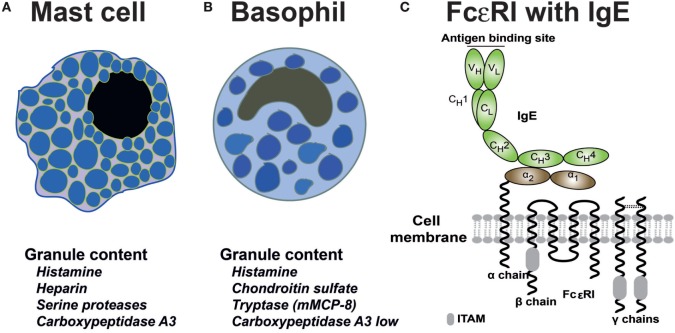
Schematic representation of mast cells, basophils and the high-affinity IgE receptor. Below the schematic pictures of the cells is in **(A,B)** a listing of the most important granule components of the two cell types. The human basophil expresses the mast cell tryptase whereas the mouse basophil expresses mMCP-8, the first basophil-specific protein to be identified (22–24). **(C)** The high-affinity receptor for IgE consists of four subunits; the α chain, which binds the CH3 domain of IgE with its membrane proximal α2 domain, the β chain that spans the membrane four times and has one immunoreceptor tyrosine-based activation motif (ITAM) in its cytoplasmic region (marked by a gray box), and two identical γ chains, which are mostly intracellular, serving as the major signal transducing subunit (25). The γ chains contain two ITAMS, one in each subunit (26–28).

Mast cells and basophils are the only two cells that express the complete high-affinity receptor for IgE (FcεRI) with all four polypeptide subunits (19) (Figures 2A–C). The FcεRI receptor belongs to a larger family of receptors binding to the constant domains of Igs, including IgG, IgA, and IgM receptors. The complete IgE receptor consists of an α chain, which is primarily located outside of the cell where it binds IgE; a β chain, which is a membrane protein with four transmembrane regions; and two identical γ chains. The γ chain has the majority of the protein on the cytoplasmic side of the membrane where it acts as the primary signal transducing subunit (Figure 2C). Other cells, such as dendritic cells and monocytes can in humans also express low levels of the IgE receptor; however in these cases, it is usually a three polypeptide variant, which lacks the β chain. This expression on dendritic cells and monocytes seems to be involved in IgE internalization and degradation, possibly also in antigen presentation (19). IgE binds to the FcεRI with high affinity, in the range of 10^10^ (20). Monomeric non-cross-linked IgE does not activate the mast cell or basophil. However, when two IgE-molecules bind the same allergen molecule they become cross-linked and if both molecules sit on the receptors, these receptors also become cross-linked. This receptor cross-linking changes the environment on the inside of the cell, favoring kinase activation at the expense of access of phosphatases to the cytoplasmic parts of the receptor subunits. This results in the phosphorylation of a number of cytoplasmic motifs on the β and the γ chains of the receptor (21). These activation motifs are called immune receptor tyrosine-based activation motifs, commonly referred to as ITAMs (Figure 2C). The phosphorylation of these short motifs result in the binding of signaling molecules including Lyn and Syk, which in turn result in a cascade of reactions including Ca^2+^ release from intracellular stores, activation of phospholipase A2 and thereby the release of arachidonic acid from membrane phospholipids and the generation of prostaglandins and leukotrienes by two different enzyme pathways. The receptor phosphorylation also results in the production of PIP3 and a few other intracellular signaling molecules as well as in the activation of the cells to release their pre-stored granule material, including histamine, heparin, and the very abundant granule proteases (Figures 2A,B).

Mast cells or mast cell-like cells have been described in most vertebrate lineages including mammals, birds, reptiles, amphibians, and bony fishes (29–32). Mast cell-like cells have also been described in an early ancestor of the vertebrates, the tunicate, or sea squirt (33, 34). Interestingly, these mast cell-like cells contain both histamine and heparin as well as some kind of trypsin-like enzyme indicating a relatively close resemblance to mammalian mast cells (33, 34).

### IgE

**Figure 3:**
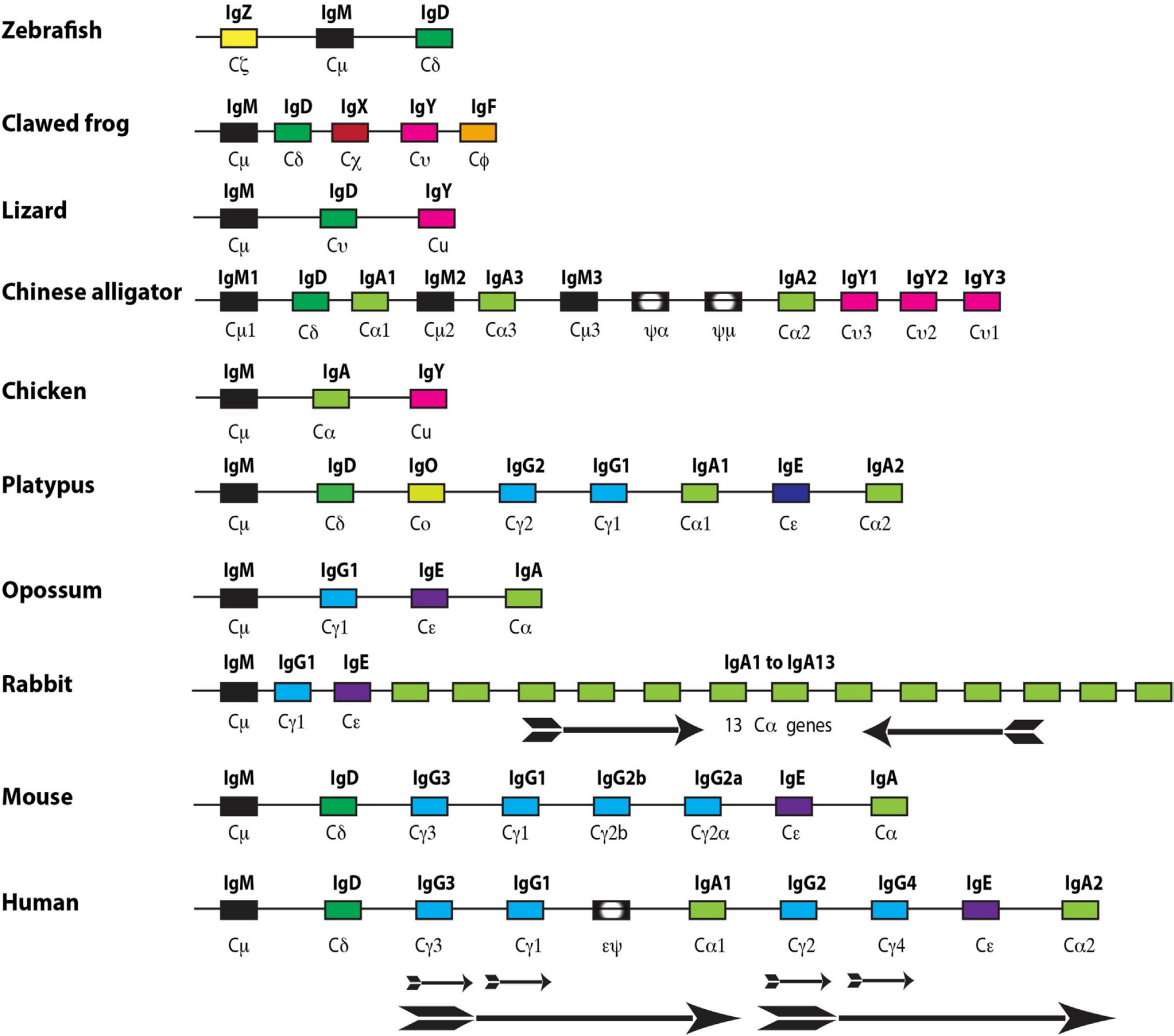
The immunoglobulin heavy chain locus from a panel of different jawed vertebrates. The different genes are color coded with IgG in light blue, IgE in purple, IgM in black, IgA in light green, IgD in dark green, IgO in greenish yellow, IgZ in yellow, IgX in red, IgY in purple, and IgF in orange. The arrows show local duplications, and the direction of the arrow represents the transcription orientation of the genes within the duplicate. Pseudo genes are marked with black boxes with a white inner oval and a pseudo sign. The figure is not to scale.

Immunoglobulins and TCRs first appeared with jawed vertebrates. There has been a parallel increase in the different vertebrate lineages in the complexity of Ig classes and isotypes during vertebrate evolution through gene duplications, which have sometimes been followed by gene losses. In general, cartilaginous fish have three Ig classes IgM and IgW, which are of the classical type with both heavy and light chains and a third IgNAR, which lacks the light chain. The entire antigen-binding site of the IgNAR thereby resides in the variable region of the heavy chain (35). Interestingly, the cartilaginous fish have a different organization of the Ig locus compared to all other vertebrates in that there are multiple genes for each Ig class and there are large numbers of such small clusters compared to single genes, which are present in most other vertebrates. The genes are organized as small rearranging units consisting of a single V, one or two D segments, followed by a single J and subsequent constant region (V-D-D-J-C). This is in marked contrast to the translocon model used by almost all other vertebrates with multiple V regions followed by a number of D segments, often over 20, and then 3-7J segments followed by constant region gene segments (35). When looking at the bony fishes, which represent the largest single group of vertebrates, there are varying numbers of Ig classes and isotypes. As one example the gar, which represent an early branch of the bony fishes, seems to primarily depend on one Ig class IgM whereas the zebra fish and the rainbow trout expresses three Ig classes, each with only one isotype, IgM, IgD, and IgT/Z (T for teleost) (Figure 3) (36, 37). Amphibians as exemplified by the clawed frog *Xenopus laevis* or *tropicalis* have five Ig classes, also here with only one isotype each, i.e., IgM, IgD, IgY, IgX, and IgF (38). IgX appears to be the functional equivalent of IgA in birds and mammals, and IgY is the ancestor of mammalian IgG and IgE (Figure 3) (38). Reptiles have similar to the bony fishes very varying numbers of Ig classes and isotypes. For example, the anolis lizard (*Anolis carolinensis*) having only three Ig classes also here with one isotype for each class, IgM, IgD, and IgY whereas the American and Chinese alligators have 4 Ig classes and 10 isotypes (Figure 3) (39–41). The alligators have experienced a series of gene duplications resulting in several functional as well as a few pseudogenes for IgM, IgA, and IgY (Figure 3). Birds have relatively few Ig classes and isotypes, probably due to later gene losses. For example, chickens have only three Ig genes, one each for IgM, IgA, and IgY (42).

In general, mammals have five Ig classes IgM, IgD, IgG, IgE, and IgA, and a varying number of isotypes. Quite big differences have been observed between different mammalian species. For example, humans have nine isotypes, due to four different IgG isotypes and two IgA isotypes. Mice have 8 due to that they have only 1 IgA isotype, and rabbits have as many as 17 because of having 13 different copies of the IgA gene (Figure 3).

**Figure 4:**
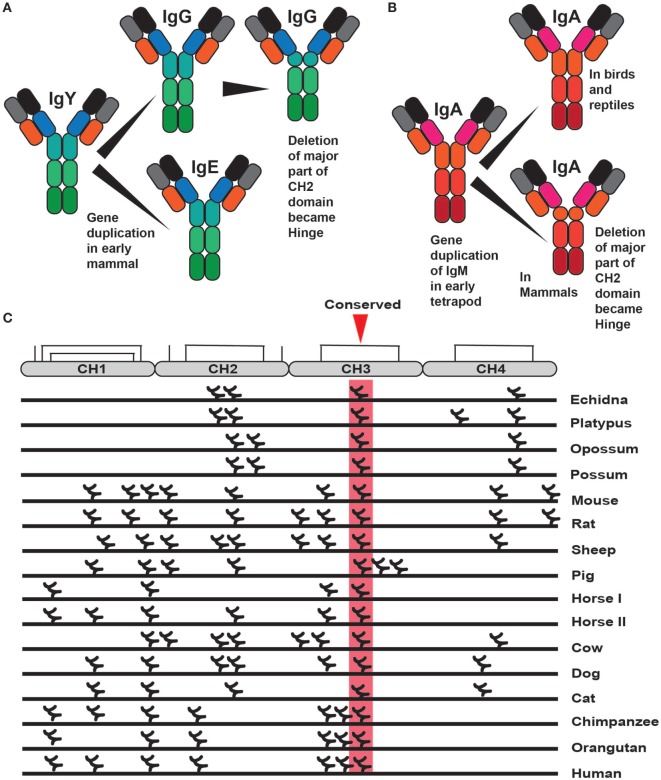
The evolution of IgE, IgG, and IgA and the distribution of N-linked carbohydrates in IgE. Panel **(A)** shows the different steps in the evolution of IgE and IgG. It began with the duplication of IgY after which IgG lost one domain, the original CH2 domain, and became an Ig with three constant domains with one hinge region (46). Panel **(B)** shows the evolution of IgA. A four constant domain IgA is present in birds and reptiles. Sometime during early mammalian evolution IgA also lost one domain, the CH2 domain, and in its place gained a hinge region (47). This hinge region is very different in size between human IgA1 and IgA2 making the former more resistant to proteases and the later more flexible, facilitating binding of several antigens simultaneously. Panel **(C)** shows the positions of the different N-linked carbohydrates in a large panel of different IgE molecules from different species. Only one carbohydrate is conserved in a position shared by all of the different IgEs, marked in red, which is positioned in the middle of the CH3 domain where it has an important function in the folding of this domain.

Interestingly, much of the functional diversification of the different Ig classes already came with the first tetrapods as the ancestors of IgA (IgX), IgG, and IgE (IgY) appeared with frogs and other amphibians. The most recent addition to the complexity came with early mammals from a gene duplication of IgY. One of the copies lost the second, CH2 encoding domain, and became a four domain Ig class with V, CH1, CH2, and CH3 domains. This gene was named IgG whereas the second duplicate, which became IgE, maintained all domains of IgY and thereby containing V, CH1, CH2, CH3, and CH4 domains (Figure 4) (43–46). Similarly to IgG, IgA also lost one domain in mammals, the CH2 domain, and instead gained a flexible hinge region (Figure 4).

IgG, IgE, and IgA are found in all three extant mammalian lineages in very similar forms (43–47). The gene duplication of IgY made it possible to separate the function of the mast cell activation *via* IgE, and the major plasma antibody, IgG, with functions in complement activation, immune complex clearance, and antibody-dependent cellular cytotoxicity (ADCC). Interestingly the marsupials, exemplified by the American opossum only have four Ig classes, IgM, IgG, IgE, and IgA, and only one isotype for each Ig class (44, 48). This is likely due to a secondary loss of IgD. The monotremes, which are an early branch on the mammalian tree with only three extant surviving members of egg-laying mammals, the platypus and two variants of the ant-eating echidnas, the short and the long nosed, have unlike the marsupials all five mammalian Ig classes as well as one additional Ig class IgO (45, 47, 49–51). The gene for IgO is most likely a remaining extra duplicate of IgY, which seems to be expressed only in the spleen at very low levels, indicating that it is primarily a non-functional remnant of the duplication process (51). The platypus has eight isotypes, IgM, IgD, IgO, IgG1, IgG2, IgE, IgA1, and IgA2. One interesting observation in mammals is that despite the very varying number of isotypes across the species there always seems to be only one functional gene for IgE (46). Currently, the only exception to this rule is within the horse, which may have two genes. This indicates that IgE needs to be kept under very stringent control, most likely due to its potent mast cell-activating properties. Interestingly, there are three copies of the IgE gene in the human genome; two of them are a result of what appears to be a large duplication involving genes for IgG, IgE, and IgA (Figure 3) (52, 53). This duplication resulted in a doubling of the genes for IgG from two to four and a doubling of the genes for IgA into two. However, following the duplication of the genes for IgE one of them has suffered a large deletion involving part of the intron and the CH1 exon to render the gene non-functional (Figure 3) (52, 53). The third IgE gene is a non-functional intron-less copy on another chromosome, which appears as an mRNA copy inserted randomly without regulatory regions (54). The selective inactivation of the duplicate gene for IgE but not IgG or IgA strengthens the indication that there is an advantage of having only one functional gene for IgE (Figure 3). It should also be noted that within eutherians, IgE has been found in all of the major branches including the orders of Primates, Rodentia, Cetartiodactyla, Lagomorpha, Carnivora, Chiroptera, Afrotherians, and Xenarthra.

One noteworthy finding concerning IgE is in the pattern of glycosylation. Almost all of the Igs are glycosylated. Most often on asparagines, so called N-linked glycosylation. The glycosylation pattern does often differ between different species and the amount of carbohydrate also correlates with the amount of positive charge (46). Therefore, if the polypeptide backbone of an Ig is highly positively charged, then there is generally a higher number of attached, N-linked carbohydrates. These carbohydrates also tend to have several negatively charged sialic acid moieties. A neutral or acidic charge makes the antibodies “less sticky,” a characteristic that may be essential for soluble effector molecules aimed to travel easily in the blood and through tissues. Of all the different carbohydrate chains of different IgGs and IgEs from different species, there is only one that is always conserved, which is in the middle of the CH3 domain of IgE (46). A carbohydrate chain is also found in the same position in the corresponding domain of IgG, the CH2 domain (46). This carbohydrate chain seems to be essential for proper domain folding and thereby for the interaction with the α chain of the IgE, and IgG receptors, which bind specifically to this domain (25, 26, 46). This indicates that with only one conserved carbohydrate position the function is to primarily to neutralize a high positive charge, where the position of the carbohydrate on the structure is of lower importance (46). Therefore, it is not the exact position rather the charge neutralization that is of importance. It also indicates that the interaction between IgE and its high-affinity receptor is a central characteristic feature of IgE, and this interaction has apparently been conserved for more than 200 years of mammalian evolution (46).

### Fc Receptors (FcRs)

**Figure 5:**
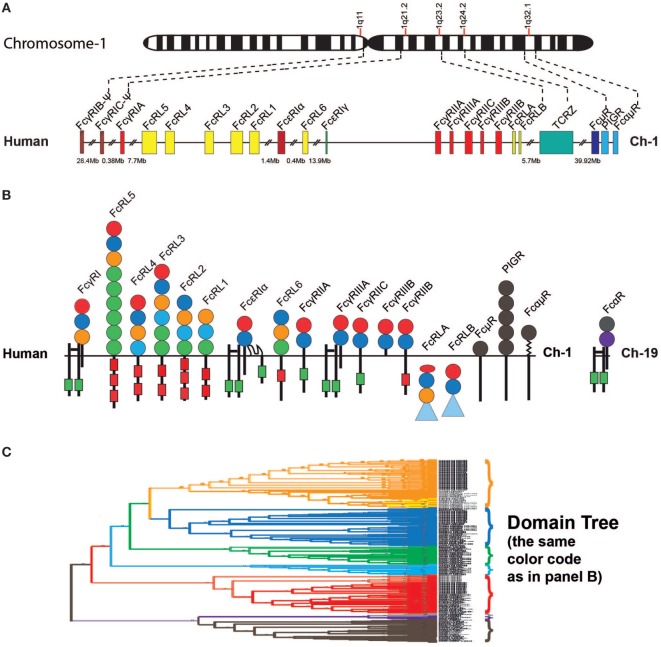
Fc receptor (FcR) genes and proteins. Panel **(A)** shows the regions within chromosome 1 in the human genome that encodes all the different FcR genes except for the specific receptor for IgA, the FcαRI, which is located together with the NK cell receptors on chromosome 19 in the humans (60, 61). Panel **(B)** shows the domain structure of the different FcRs with their different subunits. All of these receptors except FcRLA and FcRLB are transmembrane proteins. FcRLA and FcRLB are both cytoplasmic proteins. Panel **(C)** shows a phylogenetic tree of the individual domains of a panel of the FcRs, which are color coded to reflect the same subunits shown in panel **(B)**. The tree is strongly reduced in size. However, even in its reduced format, it shows the striking separation of the different domains into clearly separate branches. Only the filled circles represent the individual Ig-like domains that are included in the phylogenetic tree. The extracellular regions, the transmembrane regions, and cytoplasmic tails are not to scale in order to show the positions of potential signaling motifs such as immunoreceptor tyrosine-based activation motifs (ITAMs) (green boxes) and immunoreceptor tyrosine-based inhibitory motifs (ITIMs) (red boxes), which regulate the biological function the FcRs. Some of the intracellular proteins contain C-terminal mucin-like regions, which are depicted as blue triangles. The PIGR domains are depicted in gray.

The IgE receptor α chain is one member of a complex set of proteins interacting with the constant domains of the Igs (55–59). These molecules, named FcRs, due to their interaction with the constant domain of the Igs, have a number of important functions in vertebrates including facilitating phagocytosis by opsonization, constituting key components in ADCC as well as activating cells to release their granular content. In placental mammals there are FcRs for all Ig classes, including four major types of classical FcRs for IgG as well as one high-affinity receptor for IgE, one for both IgM and IgA, one for IgM, and one for IgA (Figures 5A,B) (60, 61). Additionally, there is the transport receptor for IgA and IgM across epithelial layers the polymeric Ig receptor or PIGR (Figures 5A,B) (61). All of these receptors are related in structure, and they all contain Ig-like domains. Furthermore they all, with the exception of the IgA receptor, are found on chromosome 1 in humans, indicating that they originate from one or a few common ancestors via successive local gene duplications (Figure 5A) (60, 61). A new family of receptors, called FcR-like (FcRL), are related in structure to the classical IgG and IgE receptors, and were discovered upon the completion of full genome sequences from a number of mammalian species (62, 63). Eight different such FcRL genes have been identified in the human genome: FcRL1–FcRL6 as well as FcRLA and FcRLB (Figures 5A,B).

All of the FcRs, including the PIGRs and the FcRL molecules, contain one or several Ig type domains. These domains have a similar fold to the Ig constant or variable domains and belong to the large family of Ig-like domains. The Ig domains have been classified into four basic types depending on the number of antiparallel beta-sheets and the positions of cysteine and other conserved amino acids. These are described as the V, C1, C2, and I type of domains (64, 65). V domains are generally found in variable regions of Igs and TCRs as well as in cluster of differentiation markers including CD2, CD4, CD80, and CD86. C1 domains are found in the constant regions of Igs, TCRs, and in MHC class I and II. C2 domains are found in CD2, CD4, CD80, VCAM, and ICAM, and I domains are found in VCAM, ICAM, NCAM, MADCAM, and numerous other diverse protein families (EMBL-EBI InterPro). All Ig domains of the FcRL and classical FcRs are classed as C2 domains, whereas the Ig domains of the PIGRs, IgM receptors (FcμRs), and IgA/IgM receptors (FcαμRs) are V type domains (64, 65). In a phylogenetic analysis of the individual domains of these receptors, the C2 and V type domains separate into clear individual branches. This is also true for the individual domains within the different receptors (Figure 5C) (60, 61).

The important signaling molecule for the classical FcRs, the common γ chain, is a member of a small family of non Ig-domain-containing molecules including the TCR zeta chain, DAP10, and DAP12 (60, 66–70). The latter two proteins serve as signaling components of NK cell receptors and as well as the related Ig-domain containing receptors (70).

**Figure 6:**
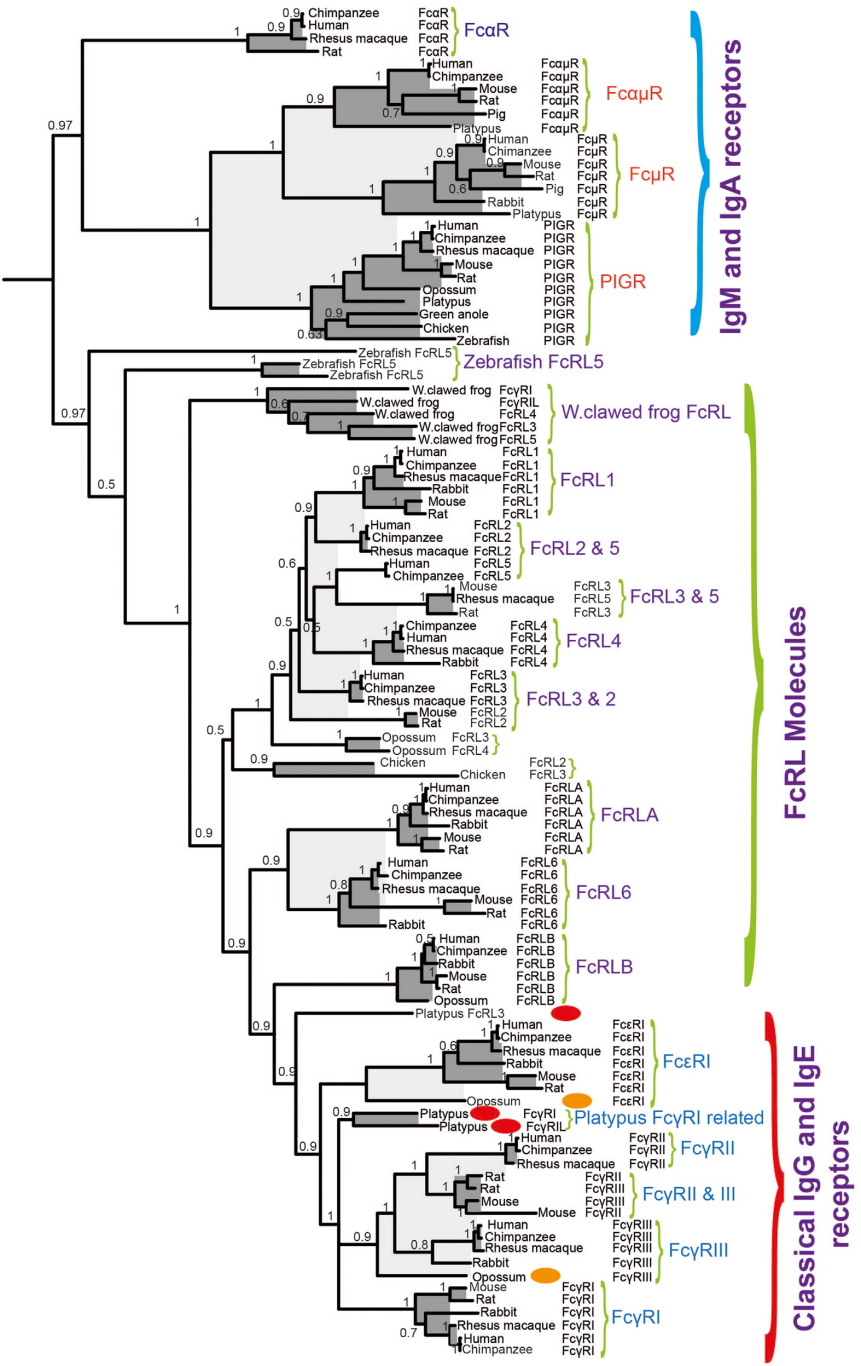
A phylogenetic tree of different Fc receptor (FcR) and FcR-like (FcRL) protein sequences. A few sequences of particular interest for the early evolution of specific IgG and IgE receptors, the discussed opossum, and platypus are marked with red and orange ovals. The Fc γ and ε sequences form a separate subfamily within the FcRL sequences, indicating that they originate as a subfamily from the FcRL genes.

Based on a phylogenetic tree (shown in Figure 6), we have found strong indications that the classical receptors for IgG and IgE likely appeared as a separate subfamily of the FcRL molecules during early mammalian evolution (60). Related genes are also found in the Western clawed frog (*Xenopus tropicalis*) and the Chinese alligator, indicating that the processes forming the subfamily of receptors that later became the classical IgG and IgE receptors may have started already during early tetrapod evolution. However, this subfamily probably did not appear as a distinct subfamily until the appearance of the mammals (60). In the Western clawed frog, these receptors have a similar structure to the human high affinity IgG receptor, FcγRI, with three extracellular Ig domains of the C2 type. In the platypus there are both two- and three-domain receptors, which are similar to the human three-domain FcγRI as well as the low-affinity IgG receptors FcγRII and III, both of which have two domains. This indicates that the development of high and low affinity receptors also took place during early mammalian evolution (60). Despite this knowledge, currently none of these amphibian, reptile, or non-placental mammalian receptors have been studied for their isotype specificities and affinities.

Interestingly, no gene related to any of the FcRs, including the PIGRs is found in cartilaginous fish. They first seem to appear with the bony fish. In bony fish, there are the PIGRs and genes closely related to the mammalian FcRL genes. There is also a gene for the signaling molecule for the classical FcRs the common γ chain, which is lacking in cartilaginous fish. This indicates a major step in evolution of the FcRs at the base of bony fish with the appearance of the PIGR, the FcR γ chain, and the FcRL molecules (60, 61). Subsequently, the second major step was the appearance of the classical receptors for IgG and IgE as a subfamily of the FcRL molecules, most likely in parallel with the duplication of IgY and the slowly emerging separation of the functions of IgG and IgE. It therefore appears to be a close evolution of the new Ig classes and their receptors.

### IgE and the Connection between Innate and Adaptive Immunity

IgE and IgG are only found in mammals and we know that in all eutherian mammals studied mast cells interact with IgE and often also IgG with isotype-specific receptors (43–45). However, Igs are found in all jawed vertebrates and mast cells are also present in most, if not all, jawed vertebrates. Therefore, one central question is when mast cells and the adaptive immunity as represented by antibodies did connect through isotype-specific FcRs. This step in the evolution is a very important step where innate immunity, which is the core of the immune system in all multicellular organisms connected with adaptive immunity. Adaptive immunity has most likely appeared a number of times and in very different shapes, which has later become an essential part of immunity in complex multicellular organisms such as the vertebrates. Although studies of the mammalian Ig repertoire and the FcR repertoires have not conclusively shown it, the results suggest that it is likely that all three extant mammalian lineages, that is, monotremes, marsupials, and placental mammals have mast cells, which are armed with high-affinity receptors for IgE (43–45, 60). However, the situation in reptiles, birds, and amphibians is much less clear. Do their mast cells have receptors for IgY? Are other isotypes involved or are there no Ig receptors on mast cells in these tetrapods?

In marsupials, represented by the American opossum, there is, based on phylogenetics, a direct ortholog of the human and mouse IgE receptor α chain (Figure 6) (60). Although it is most likely that this receptor binds IgE, it has not yet been proven. The situation in the monotremes, represented by the platypus, is even less clear, although there are receptors in the platypus genome, which are closely related to the IgG and IgE receptors in placental mammals (Figure 6). The two receptors that are most closely related to the IgG and IgE receptors in placental mammals appear as a separate branch in-between the IgG and IgE receptors in the phylogenetic three (Figure 6) (60). The isotype specificities of these two receptors and the single receptor, which is located just outside of the IgG and IgE receptors in the tree have not yet been studied but it is reasonable to think that one of them is IgE specific and the other is IgG specific. There are no direct homologs to the IgG and IgE receptors in birds and reptiles. However in the clawed frog, *Xenopus laevis*, there are three genes, which may be a very early ancestor to the IgG and IgE receptors (Figure 6) (60). However in a similar manner to the platypus, there is not yet any information on their isotype specificity, therefore we do not know if they bind IgY, IgX, IgM, or IgF or perhaps none of them.

### Mast Cell and Basophil Granule Components

Both mast cells and basophils contain a large number of cytoplasmic granules (Figures 2A,B). These granules that are functionally related to lysosomes, store a number of substances of low and high molecular weight substances. Histamine, one of these low molecular weight compounds, is stored by both mast cells and basophils (71). It is based on the amino acid histidine where the carboxyl acid, i.e., COOH group, has been removed by the enzyme histidine decarboxylase (72). The removal of the acid group results in a positively charged molecule of a size smaller than that of an amino acid. Histamine is a highly potent inflammatory mediator due to its interaction with four different receptors termed H1, H2, H3, and H4 (73, 74). Binding of histamine to these receptors induces a number of processes including vascular leakage and itching (73, 74). The mast cell granules also contain large and heavily sulfated, negatively charged polysaccharides; heparin in the case of mast cells, and chondroitin sulfate in basophils (75–78). One function of these charged proteoglycans is most likely to counteract the positive charge of histamine. The cell would otherwise not be able to store such large amounts of a positively charged molecule (77). Heparin is also a potent anticoagulant *via* its binding and activation of anti-thrombin (78).

Mast cells also store massive amounts of proteases primarily chymotrypsin/trypsin-related serine proteases but also the mast cell-specific carboxypeptidase A3 (79–85). These proteases can make up to 35% of the total protein, thereby constituting the absolute majority of the protein content of the mast cell granules (86). Most of these proteases are positively charged and therefore bind to the long, negatively charged proteoglycan polysaccharide chains. Tryptases are one subfamily of serine proteases, which are also dependent on heparin for maximal activity (87). The mast cell tryptases form tetramers where the dependency on heparin keeps these formations together, as well as increasing their proteolytic activity (88, 89). Basophils also express proteases but to a much lesser extent. Human basophils primarily express the tryptase whereas mouse basophils express the basophil-specific protease mMCP-8 (22–24).

**Figure 7:**
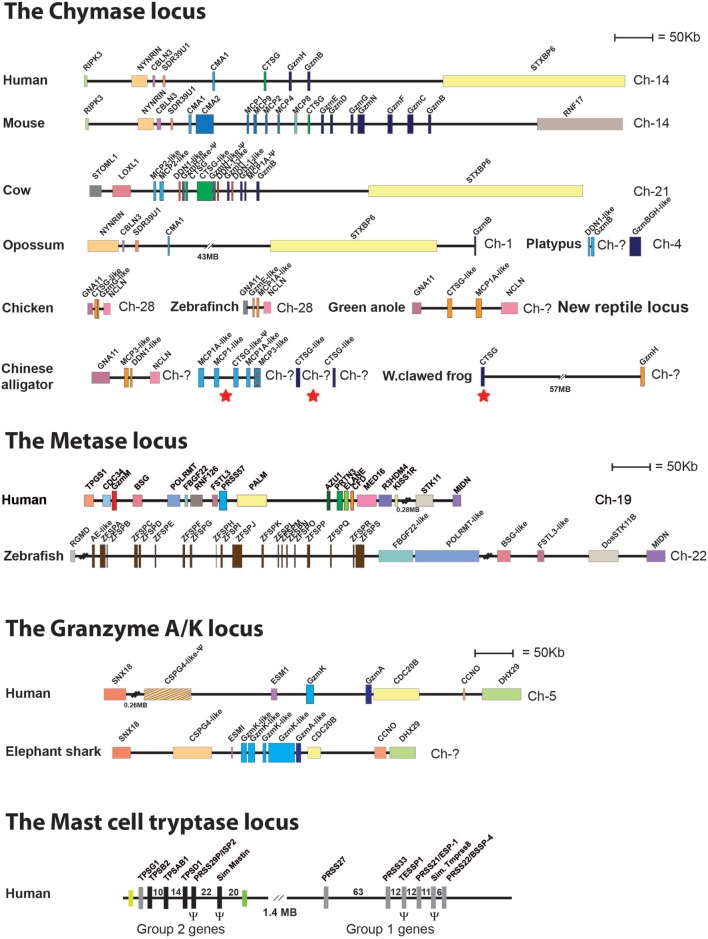
Chromosomal loci encoding hematopoietic serine proteases. A selection of such loci representing the five different loci encoding hematopoietic serine proteases is shown; the chymase locus, the metase locus, the granzyme A/K locus, the mast cell tryptase locus, and the new chymase locus related locus found in amphibians, reptiles, and birds. The section of the figure showing the chymase loci includes this new amphibian, bird, and reptile locus. These chymase locus related genes are marked in orange. A number of bordering genes are also included to show the similarity in the surrounding regions between the different species. The loci or genes marked with red stars in the alligator and frog genes (under chymase locus genes) are the genes closely related to the mammalian chymase locus genes and thereby may represent an early variant of the mammalian chymase locus. A more detailed analysis of these protease genes and their evolution containing a much larger number of species is found in Akula et al. (90).

One of the major questions in the field is the function of these very abundant proteases. To trace the origin of these proteases and thereby get additional clues to their conserved primary functions, we have performed several evolutionary studies of the different loci encoding the different mast cell and basophil proteases (85, 90). These proteases belong to a larger subfamily of related proteases that are expressed by a number of hematopoietic cells and have therefore been named hematopoietic serine proteases. They are expressed by mammalian mast cells, basophils, neutrophils, CTLs, and NK cells. In mammals, these serine proteases are encoded from four loci (85, 90–92). One additional locus with related proteases is found in reptiles, indicating a loss of one locus in mammals (90). In mammals, the four loci include the chymase locus, which in humans encodes the mast cell chymotryptic enzyme, the chymase; the neutrophil cathepsin G; and a few T cell- and NK cell-expressed granzymes (Figure 7). In humans, only two granzymes are present in this locus, granzyme B and H (Figure 7). A massive expansion of the chymase locus both in size and number of functional genes has occurred in rodents. The mouse chymase locus has, for example, 15 functional genes, including two new classes of genes, the β-chymases and the mMCP-8 gene and also several additional granzyme genes (Figure 7) (85, 90). The rat locus is fifteen times larger than the dog chymase locus and contains 28 functional genes (85). In ruminants, i.e., cows and sheep, an additional subfamily called the duodenases has appeared *via* gene duplications, most likely from the granzymes or from cathepsin G (85, 90). The duodenases in cow have changed tissue specificity and are now not expressed in hematopoietic cells but in secretory cells in the duodenum, where they most likely take part in food digestion (93, 94). The second locus is the metase locus, which encodes most of the neutrophil proteases; *N*-elastase, proteinase-3, neutrophil serine protease-4, and an inactive variant, azurocidine that acts as an antibacterial substance without protease activity. This locus also encodes a complement component, complement component D (Figure 7) (85, 90). The third locus encodes two T-cell expressed proteases, granzyme A and K, which are both tryptic enzymes (Figure 7). The fourth locus is the mast cell tryptase locus, which is divided into two regions, one expressing primarily the mast cell-expressed soluble tryptases and the second, which primarily encodes membrane bound tryptases that show a relatively broad pattern of expression (Figure 7) (95–97). The fifth locus has completely different bordering genes and is only found in reptiles, birds, and amphibians, where it encodes a few proteases that are distantly related to the mammalian chymase locus genes (Figure 7) (85, 90). By looking into the genomes of a panel of vertebrates, it has been possible to trace the origin of these genes during vertebrate evolution. There is evidence for the existence of the T cell tryptase locus, encoding granzymes A and K, from cartilaginous fish to humans but not in jawless fish, including the lamprey and hagfish. Similarly, this locus is not found in tunicates or echinoderms, indicating an appearance with early jawed vertebrates. This locus is the only one of the five loci that is found in cartilaginous fish, indicating that it is the oldest of them all (Figure 7) (85, 90). In bony fish, there is evidence for the presence of the metase locus, and in frogs, the first gene that can be directly seen as an ancestor of the mammalian chymase locus is found. In frogs, a gene for the fifth locus exists, which is present in reptiles and birds but not in mammals. In this manner, we can start to see a gradual and probably parallel appearance of these proteases in the different vertebrate lineages during vertebrate evolution (85, 90).

In order to obtain additional functional information concerning these proteases, we have used an unbiased technique with a very large number of potential target sequences. This consists of a phage library with 50 million different 9 amino acid long random sequences to study the extended cleavage specificity of a number of these hematopoietic serine proteases (85, 98).

**Figure 8:**
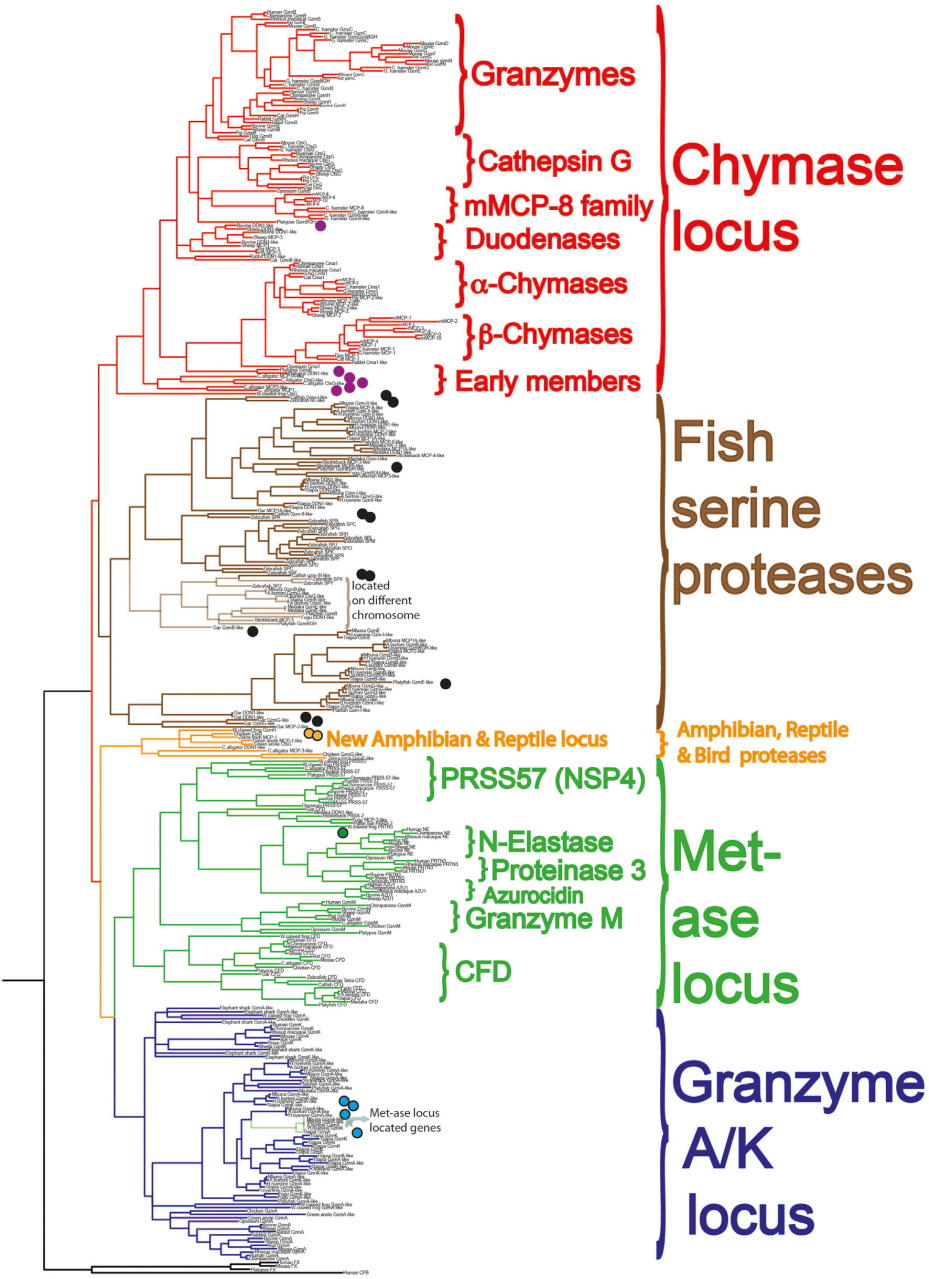
A phylogenetic analysis of a large panel of hematopoietic serine proteases. Three coagulation and complement components are used as outgroup. The proteases of the different genetic loci cluster together in separate branches of the tree and are color coded in similar fashion to Figure 7: the metase locus genes marked in green, the granzyme A/K locus in dark blue, the new reptile and amphibian locus in orange, the chymase locus marked in red in one branch, and the majority of the fish proteases in brown. The proteases we are currently analyzing for their extended cleavage specificities and tissue or cell type expression patterns are marked with small filled circles. A more detailed analysis of these protease genes and their evolution is found in Akula et al. (90).

Using a combination of genomic analyses and functional analyses of the extended cleavage specificities of a selected panel of the proteases, we have started to get a more detailed view of their emergence, evolution, cleavage specificities, and the basic functions performed by these proteases (22, 23, 80, 82, 85, 90, 98–106). The proteases we have focused on are the most interesting ones from an evolutionary perspective, i.e., ones that represent major branches on the phylogenetic tree and that can be most easily studied for their *in vivo* function based on available model systems (Figure 8) (85, 90).

Both chymotryptic and tryptic enzymes appear to have been a central component of mast cell granules from early mammals. This is based solely on the presence of the protease genes in marsupials and monotremes and not on a direct analysis of their mast cells. However, based on the presence of proteases with close structural similarities and with very similar cleavage specificities to the well-characterized ones in several placental animals, we can with reasonable certainty claim that they are also likely to be part of the mast cell phenotype (90, 103). The picture is less clear when we look at reptiles, birds, and amphibians. In birds, none of the classical chymase locus genes are present and in frogs, there is one such gene, which has structural similarity of its active site pocket that closely matches one of the T cell enzymes, granzyme B (Figure 7) (90). We have now also started to study the related fish proteases (Figure 8, brown colored branch) (90, 107), and have so far produced recombinant protein for three catfish proteases. Two of them are expressed in CTLs or NK-like cells and one from a macrophage-like cell line. One of these proteases, catfish granzyme-like I is a highly specific protease, probably the serine proteases with the highest specificity characterized so far. The cleavage specificity of this protease, which is expressed by fish NK-like cells, has an ability *in vitro* to cleave a sequence within catfish caspase 6, indicating it may have similar function as mammalian granzyme B, thereby inducing apoptosis in target cells (108). The second catfish protease is a highly specific tryptase, as of yet an unknown function, which is expressed by fish macrophage-like cells (unpublished results). The third of these three catfish proteases is not yet characterized at all and we do not know its specificity (90, 107). Several other fish proteases have also been produced as recombinant proteins, including ones from gar, zebrafish, and platyfish (Figure 8). However, no information concerning their tissue specificities, cleavage specificities, or potential targets are known yet; therefore currently, we cannot say if these fish proteases show similarities to any of the mast cell proteases in mammals. These studies are in their infancy and hopefully a more detailed picture will emerge within a few years’ time.

Despite numerous studies concerning the major functions and major targets of mammalian hematopoietic serine proteases, the picture concerning these proteases is still relatively incomplete. An array of potential targets has been described, some more and some less likely to represent evolutionary conserved functions of these proteases.

The five most interesting and in our minds most logical roles for the mast cell proteases include the following: they most likely have a central role in the defense against various snake, scorpion, and bee venoms (109–111). There is sufficient evidence that they probably also take an active part in connective tissue remodeling by cleaving connective tissue components, as for example, fibronectin, and/or by activating other proteases, for example, matrix metalloproteases (MMPs), which can degrade several components including collagen (112–117). In addition, they are likely involved in both activation and degradation of cytokines, primarily inflammatory cytokines to dampen inflammation (106, 118). The mast cell chymase is also a potent activator of angiotensin from Ang I to Ang II thereby with the potential to increase blood pressure, which may be needed after a systemic mast cell activation where blood pressure drops due to the out-flow of liquid from the blood into the surrounding tissue (117, 119–121). During the opening of blood vessels to enhance the flow of blood components including antibodies and complement, there is a major risk that the inflow is blocked by coagulation. Here, mast cell proteases and heparin may act cooperatively as an anticoagulant by cleaving thrombin and other coagulation components (114, 122, 123). Although not to be ignored, there are many other suggested functions. However, given the evidence, we feel that these five are among the most likely. These proteases may have adapted a relatively broad array of functions, which may also explain their relatively broad specificities.

### What Is the Physiological Role of IgE?

One of the major questions in the field of IgE biology is why this gene that on face value causes us so much trouble has been maintained for several hundred million years of mammalian evolution. The indications for a coevolution of IgE and its receptors on mast cells and basophils strongly support an evolutionary selective advantage of the system. During the writing of my PhD thesis in 1985 I (LH) proposed a theory that IgE together with mast cells may actually function as a door or gate keeper, stopping the antigen at the site of entry (124). Much of the evidence that has accumulated on the subject now strengthens this hypothesis. By triggering a rapid release of histamine, prostaglandins, leukotrienes, proteoglycans, proteases, and cytokines mast cells can activate and recruit immune cells to the site of entry. Histamine and the arachidonic acid metabolites (leukotrienes and prostaglandins) open blood vessels, which facilitates the entry of antibodies, complement and immune cells. The proteases and heparin that are also being released by the mast cells can limit coagulation, which would otherwise inhibit the movement of inflammatory cells. The proteases can also function by loosening up the connective tissue allowing the entry of the immune cells and other molecules. The location of mast cells in regions where most pathogens enter, for example, the intestinal and lung mucosa, the skin as well as around organs and blood vessels, also provides strong support for the role of IgE and mast cells as part of a door keeper or sentry function. At these sites mast cells can also exert an important role in the process of venom inactivation. Likewise at these locations, the processing of Ang I into Ang II by the mast cell chymase can potentially counteract the resulting effect of the blood pressure drop after mast cell degranulation. Release of Ang II can result in a rapid increase in blood pressure. The tissue remodeling function of mast cells by activation of MMPs and cleavage of fibronectin, collagen, and other connective tissue components is probably relatively IgE independent, whereas cytokine activation or inactivation may be important for limiting the inflammation initiated by the IgE-dependent mast cell activation. We have also observed a peculiar early IgE response already at days 3 and 4 after antigen/allergen contact that seems to precede the rise in IgG (unpublished observations). IgE responses have also been seen to occur primarily against low levels of antigen. This would indicate that the IgE system is focused on early responses to low levels of antigen, possibly to sample the environment. The antigen-specific IgE, often locally produced, can then bind mast cells and prime the immune system for a second encounter with this antigen possibly in the form of a parasite, a virus, or a bacteria. The individual is subsequently already primed for a relatively strong response involving the majority of immune components. The IgE covered mast cell is an extremely potent amplifier of an inflammatory response, where cross-linking of less than 100 IgE molecules on the cell surface is sufficient for full activation/degranulation of the cell. Such a sensitive and massive response may be necessary to manage a massive infection of an intestinal worm parasite. Therefore, in our minds many aspects related to IgE and mast cells, including their location and homeostasis, favor a role of these components in an early, door keeper function.

### IgE Levels under Parasite-Free and Parasite-Rich Conditions

Non-allergic persons have generally very low levels of IgE in their circulation, ranging from 20 to 400 ng/ml in blood (125). In comparison to the IgG levels, which range from 8 to 16 mg/ml, IgE levels are between 100,000 times to a million times lower. Persons with the relatively mild allergies, rhinitis, and conjunctivitis, often have slightly elevated IgE levels, where asthmatics have even higher ranging from 400 ng to 1 or 2 μg per ml. The patients with the highest IgE levels are generally persons with severe atopic dermatitis where IgE levels may reach as high as 10 μg/ml.

An interesting question here is if these very low levels of IgE are reflected in the general situation in both domestic and wild animal populations, and how this affects our view of the function of IgE. Most inbred mouse and rat strains have similar low IgE levels to non-allergic humans, below 200 ng/ml, with many strains having less than 50 ng/ml (7, 126). However, there are some notable exceptions with strains such as the Balb/c mice, which may reach 100 μg of IgE/ml as well as the Brown Norway rats, which can have IgE in the range of several micrograms per milliliter (7). Both of these rodent strains are considered so called TH2 type of strains, with a dominance of humoral immunity compared to many strains with low IgE levels that are more TH1 prone, thereby having a stronger tendency to use cell-mediated immunity. Therefore, genetic factors are clearly important for the levels of circulating IgE.

However, other factors are also very significant. Both humans and rodents living under laboratory conditions are generally free from worm infections, which are known to be potent inducers of IgE production (127). By contrast, most wild animal populations have massive amounts of intestinal worm parasites.

**Figure 9:**
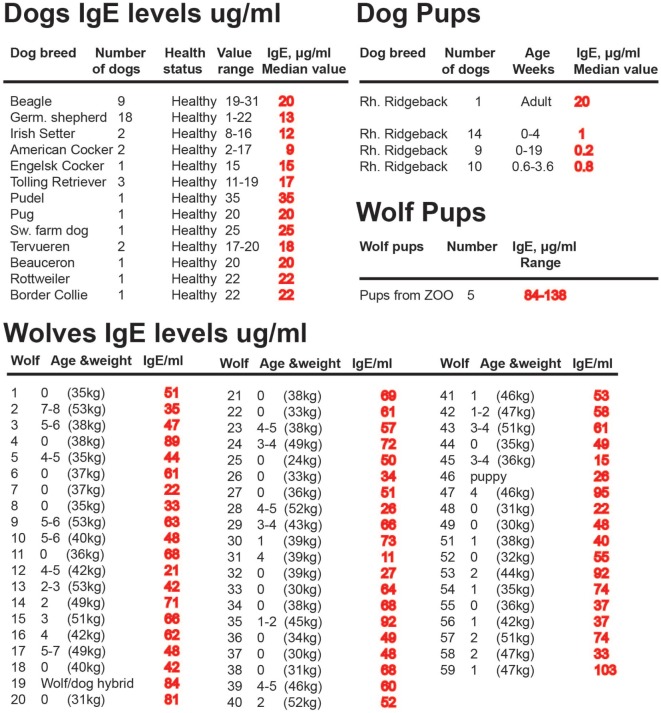
IgE levels in a panel of domestic dogs and wild wolves. The IgE levels, in micrograms per milliliter, are marked in red after each animal or group of animals. Values from 33 young dogs and 5 young wolves are also included in the figure. The young wolves were from a zoo due to the difficulty obtaining samples from wild wolf pups.

A few years ago we developed a reliable assay testing for dog IgE based on monoclonals raised against recombinant dog IgE. This made it possible to study IgE levels in both dogs and wolves, with high accuracy, which had not been possible with previously existing reagents. Analysis of a panel of 76 adult dogs showed that adult dogs have IgE levels that are between 10 and 40 μg/ml, which is almost 100 times higher than non-allergic humans, but that young dogs started with low with levels often below 1 μg/ml of IgE (Figure 9) (128). A collaboration with Professors Jon Arnemo and Olof Liberg, which involved a large interdisciplinary study on the Scandinavian wolves, Scandulv, made it possible to obtain serum samples from approximately 30% of the total Scandinavian wolf population, around 65 individuals. By analyzing their IgE levels, we could see that wolves in general have twice as high IgE levels compared to domestic dogs, having a median value of 67 μg/ml (Figure 9) (129). The relatively high levels seen in domestic dogs (compared to non-allergic humans) were somewhat surprising as they are a domestic population. However, dogs are frequently parasite infected, then undergo treatment for this, but often get re-infected. This indicates that after infection levels of IgE tends to stay high for relatively long periods of time. A similar situation is seen with horses. Domestic horses have been found to have very high levels of IgE, twice the levels seen in wolves somewhere between 30 and 180 μg/ml (130). Horses also often get re-infected with worm parasites when grazing, which means they tend to need repeated treatment for intestinal worm infections (131, 132). Analysis of the young dogs previously described showed relatively low IgE levels, below 1 μg, indicating that parasite infections may be the major cause of the high IgE levels seen in adult dogs (Figure 9) (128). This also suggests that the puppies stay uninfected until they start to go outside their homes. Interestingly by contrast, the few young wolves coming from a zoo that were analyzed showed even higher IgE levels than the adult wolves, possibly indicating an early parasite infection or a genetic difference in IgE regulation between dogs and wolves (Figure 9). During the cloning and analysis of Ig isotypes in the platypus, we also found that transcript levels for IgE in the spleen were only six times lower than the IgG levels of these free-living animals, which further support the finding that IgE levels are much higher in wild compared to domestic animals (45).

A very interesting study of Ethiopian Jews has also been performed, which provides indications further in line with these animal findings. When IgE levels were analyzed on people that had moved from Ethiopia to Israel at an adult age their IgE levels were high and stayed high during the entire study, whereas their children who were born and raised in Israel had similar low levels of IgE as other children born in Israel (125). The situation seen in dogs, horses, platypus and the migrating Ethiopian Jews show that IgE levels tend to stay high for long periods of time even after being free from parasite infection. The reasons for this are not known but indicates that the cytokine environment may change more permanently after a long or repeated exposure to intestinal worm parasites (133).

### Domestication and the Appearance of Allergies

A number of factors have been indicated to be of importance for the high incidence of allergies in industrialized countries. One factor that seems to have a major impact is general domestication, as it is primarily among ourselves and among domestic animals that we find allergic individuals. To our knowledge, allergies have not been described in wild animals. One potential factor could be a genetic drift due to strong selection for phenotypic characteristics like coat color, long or short noses, running fast, or wanted social behaviors. Such strong selections are seen in the breeding programs for dogs, horses, and cats, but a questionable cause for human allergies. However, it is possible that we constantly need to be selecting against hypersensitivities, which may occur due to minor shift in immune functions caused by spontaneous point mutations. A strong such selection process most likely exists in wild animals under tough environmental conditions but not in domestic animals and in humans. Another factor could be parasite infections. However, the presence or absence of parasites cannot be the only explanation as we see allergies both in ourselves, who are generally free from intestinal parasites, at least in the industrialized world, whereas dogs and horses are often parasite infected.

There could also be numerous other contributing factors, which are only partly dependent on domestication such as hygiene including a reduced complexity of intestinal microbiome. For humans, one factor could be the reduced use of fermented food. The list has become relatively long for potential causes of this increase and there are studies, which favor and disfavor almost all of them, making the situation very complex. It is clear that not only one single factor is involved but a combination of many and often also diverse factors, which together provides the imbalance of the immune system to overreact against often harmless substances such as pollen, nuts, animal fur, cow milk proteins, eggs, fish, and shellfish.

### What We Can Learn from Wild Animals

By studying only a few species, which live under very similar conditions we most likely get a fairly limited view of the regulation of the immune system. For example, many of the knockout strains of mice have been shown to display a very mild phenotype, indicating that many cell types and molecules are dispensable and not essential. However, this may be the situation under low pathogen loads and under conditions of good access to nutrients and clean water. A more pronounced phenotype may be seen under stress, under conditions of limited water and food supply, when individuals have to fight for territory and mating partners and under heavy parasite loads. Similarly, we may not see the factors regulating a normal immune response by only looking at a few species, which are essentially living under very similar and limited environmental conditions, including absence of parasites, a lack of fighting for territory, and an almost unlimited supply of food and water. One interesting finding relating to this idea is that persons lacking IgE seem to live a relatively normal life in spite of the fact that the connection between IgE and mast cells has been evolutionary conserved for what it seems at least 200 million years (134). By studying wild animals under natural conditions, we may get a better picture of the factors that have shaped our immune system. Recent data from the currently rapidly evolving field concerning the role of the microbiome show that as we are eating the same foods with low amount of fiber and high fat or high sugar, this can reduce the complexity of the intestinal flora (135, 136). A similar effect may also come from a massive overuse of antibiotics (137). A diverse microbiome obtained from eating different foods and not using antibiotics may be one factor in this picture (126, 138, 139). We know that the microbiome is important for stimulating the immune system, and that microbes are important for the production of vitamins and for the degradation of hormones and other substances. This may be suggestive as to why a reduced food complexity may be one factor that limits the development and functional diversity of our immune system. We are bombarded by the idea that lactobacilli, for example, are beneficial for our health. However, numerous trials with probiotics, including eating live lactobacilli in the form of yogurt or fermented foods have not shown any significant effects [reviewed in Ref. (140)]. Here the problems may be partly related to that these added lactobacilli only appearing to live for a short period of time in the intestine, thereby only marginally affecting the intestinal mucosa and the immune cells residing in the area just under the epithelial cells. In the industrialized world, we are almost completely free of both ecto and intestinal parasites. This is a factor that we as species are not so well adapted for. For example, studying the intestines of wild mice reveals that they are typically full of intestinal parasites, yet interestingly they are otherwise in reasonable health. Similarly, they are usually also infested with ecto-parasites including lice, fleas, and ticks factors that may markedly affect the immune system by triggering inflammatory cells, thereby affecting the cytokine environment. Although these factors may be of importance for the development of our immune system we do not perceive that anyone of us would like to return to such conditions. However, the lesson here is that factors such as parasite loads and complexity of the intestinal flora may be of importance for many diseases including allergy, autoimmunity, diabetes, and possibly even certain cancers (127, 141, 142). Here studies of wild animals can shed light upon how the immune system reacts to, and handles, all of these parasites and also how a diverse intestinal flora may affect these processes. Respiratory virus infections are most likely also an important factor where respiratory syncytial virus and rhino virus are of prime interest for asthma development (143). There are other, more discrete factors, for example, there is an ever increasing amount of hormone-like substances in our environment, such as estrogens from contraceptives that enter the sewage system. These may also be other factors to take into account, as it is well known that such substances can markedly affect reproduction of local fish and amphibian species. Wild animals are often more exposed to such pollutants than us, for example, fish and amphibians can live directly within the polluted water and thereby take up the substances more effectively. Studies of such animals in the wild can provide information on how such pollutants affect their immune functions and thereby give us clues to as to how these substances will also affect us. Such substances may affect us more subtly by shifting the balance of the immune system. The question is very complex, and here by looking at wild populations may present us with a more multifaceted view of the factors involved in shaping immunity and where these factors can go wrong. Our life style has changed dramatically, and the strong genetic selection acting upon us during early evolution is presently most likely not as efficient, which may be contributing factors to the increase in allergies. However, what factors that dominate this increase are still not known, which may also vary from person to person as both genetic and environmental factors seem to be of importance (127, 140).

### Wild Animals As a Source of Therapeutic Proteins

**Figure 10:**
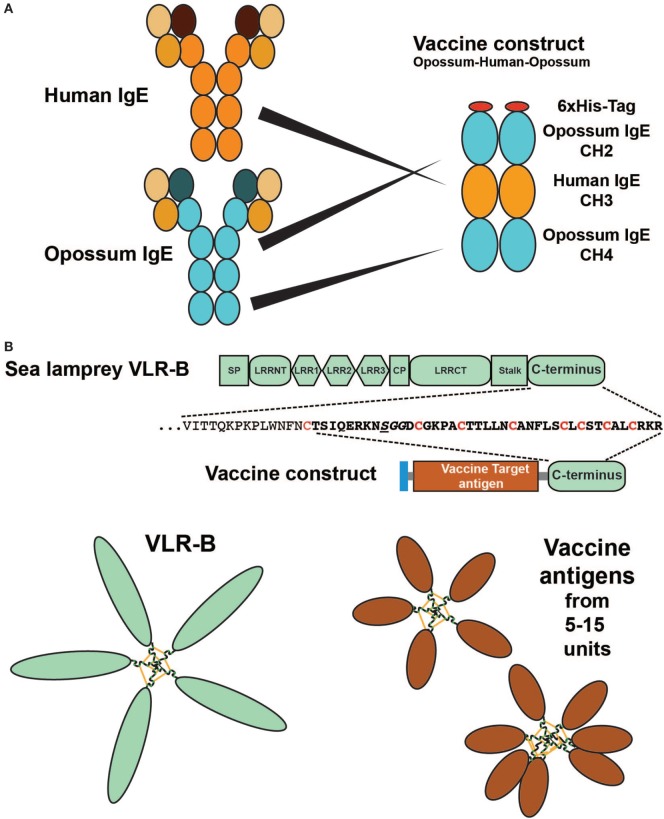
Therapeutic vaccine proteins where parts of the vaccine antigen originate from a wild animal. Panel **(A)** shows a vaccine antigen consisting of a fusion protein between the CH2 and CH4 domains of opossum IgE together with the target region for the vaccine: the CH3 domain from the target animal, a human, dog, or rat. The figure shows the human variant of the vaccine. The rat variant of the vaccine antigen has been shown to induce a strong anti-self-IgE response in rats of several strains and to reduce circulating IgE levels in these animals (7). Panel **(B)** shows a multimeric vaccine component generated by the use of the C terminal tail of the lamprey antigen-specific receptor variable leukocyte receptor B (VLR-B). VLR-B is the functional but not the structural equivalent of human IgM, a pentameric antigen-binding molecule. Using the C terminal 30–40 amino acids from VLR-B fused to the C terminal of any soluble vaccine antigen, it is possible to obtain a multimeric vaccine antigen that serves as a very potent antigen due to their similarity to virus particles or bacterial surfaces with multiple identical epitopes. Using three cancer vaccine antigens as test antigens, we have shown that they are soluble when produced in bacteria (*E. coli*) and form stable cysteine-bridged multimers with from 5 to at least 15 monomers, in the multimeric structures (150).

In addition to giving us a more detailed view of the regulation and the evolution of our immune system, wild animals may also be a rich source of therapeutic proteins and other potential therapeutic molecules. As a separate line of research we have also been trying to develop new treatment strategies against atopic allergies. One such line of research has been the development of therapeutic vaccines targeting IgE and several of the early TH2 inducing cytokines including IL-33, IL-18, and TSLP (Figure 1) (6–8, 13, 144). Induction of an immune response against self-molecules, as is the case for all of these targets, is considerably more difficult than inducing an immune response against a foreign molecule. We are generally tolerant to self-molecules, and therefore, we need to use a number of tricks to overcome these tolerance mechanisms in order to induce an immune response strong enough to give a therapeutic effect. Here, adjuvants are very important and in order to obtain a strong anti-self-immune response potent adjuvants are a necessity (145, 146). On top of this issue are other factors including having to modify the self-protein by coupling it to a non-self carrier (6, 144, 147). This results in the recruitment of non-tolerized T cells to provide help to self-reactive B cells to expand and differentiate (144). In the design of a vaccine targeting IgE, we produced a fusion protein between opossum IgE and human, rat or dog IgE (Figure 10A). The rat variant of this vaccine antigen induced a strong anti-rat IgE response in sensitized rats and resulted in a marked reduction in circulating IgE titers in these animals (7). Another important factor is the form the self-antigen is being presented (148). Multimeric antigens such as a virus particle or a bacterial surface are very potent antigens, probably due to their potent B cell-activating properties (148, 149). A multimeric antigen can crosslink IgM on the surface of the naïve B cell very efficiently and thereby giving a very strong activating signal 1 to the B cell. During the process of optimizing parameters to obtain potent therapeutic vaccines against allergies and different solid cancers, we identified a region of a molecule coming from a wild animal a jawless fish. The protein and region is the tail-piece of the variable leukocyte receptor B (VLR-B) that facilitates the pentamerization of the VLRB, which is the functional but not structural Ig equivalent in lamprey and hagfish, similar to pentameric human IgM. Using the 30 amino acid C-terminal region of lamprey VLR-B, this resulted in very efficient multimerization of the target antigen and in a marked enhancement of the anti-target immune response (Figure 10B) (150). In this context, wild animals can not only give us a more detailed view of the function and evolution of our immune system but can also be a rich source of potential therapeutic proteins.

## Conclusion

Wild animals can teach us a lot about our own immune system, including how it is regulated, how it has evolved, and which functions are essential for a potent immune defense. These reasons are but a few to consider, where non-domestic animals may facilitate new solutions to difficult therapeutic challenges.

## Author Contributions

The review was co-written by LH, SA, MT, and ZF.

## Conflict of Interest Statement

The patents for the VLR-B sequence used to enhance immunogenicity to therapeutic vaccines targeting self-molecules is owned by a company owned by LH. All other authors declare that the research was conducted in the absence of any commercial or financial relationships that could be construed as a potential conflict of interest.
